# Monitoring Sub-Saharan African Physician Migration and Recruitment Post-Adoption of the WHO Code of Practice: Temporal and Geographic Patterns in the United States

**DOI:** 10.1371/journal.pone.0124734

**Published:** 2015-04-13

**Authors:** Akhenaten Benjamin Siankam Tankwanchi, Sten H. Vermund, Douglas D. Perkins

**Affiliations:** 1 Program in Community Research and Action, Department of Human and Organizational Development, Peabody College of Education and Human Development, Vanderbilt University, Nashville, Tennessee, United States of America; 2 Vanderbilt Institute for Global Health and Department of Pediatrics, Vanderbilt University School of Medicine, Nashville, Tennessee, United States of America; Johns Hopkins University, UNITED STATES

## Abstract

Data monitoring is a key recommendation of the WHO Global Code of Practice on the International Recruitment of Health Personnel, a global framework adopted in May 2010 to address health workforce retention in resource-limited countries and the ethics of international migration. Using data on African-born and African-educated physicians in the 2013 American Medical Association Physician Masterfile (AMA Masterfile), we monitored Sub-Saharan African (SSA) physician recruitment into the physician workforce of the United States (US) post-adoption of the WHO Code of Practice. From the observed data, we projected to 2015 with linear regression, and we mapped migrant physicians’ locations using GPS Visualizer and ArcGIS. The 2013 AMA Masterfile identified 11,787 active SSA-origin physicians, representing barely 1.3% (11,787/940,456) of the 2013 US physician workforce, but exceeding the total number of physicians reported by WHO in 34 SSA countries (N = 11,519). We estimated that 15.7% (1,849/11,787) entered the US physician workforce after the Code of Practice was adopted. Compared to pre-Code estimates from 2002 (N = 7,830) and 2010 (N = 9,938), the annual admission rate of SSA émigrés into the US physician workforce is increasing. This increase is due in large part to the growing number of SSA-born physicians attending medical schools outside SSA, representing a trend towards younger migrants. Projection estimates suggest that there will be 12,846 SSA migrant physicians in the US physician workforce in 2015, and over 2,900 of them will be post-Code recruits. Most SSA migrant physicians are locating to large urban US areas where physician densities are already the highest. The Code of Practice has not slowed the SSA-to-US physician migration. To stem the physician “brain drain”, it is essential to incentivize professional practice in SSA and diminish the appeal of US migration with bolder interventions targeting primarily early-career (age ≤ 35) SSA physicians.

## Introduction

Universal health coverage, a priority goal for the World Health Organization (WHO), cannot be achieved without “an adequate, skilled, and motivated health workforce working within a robust health system” [1–2]. In the WHO Africa region, most of which comprises International Monetary Fund-designated “heavily indebted poor countries” [3], the health worker crisis has been compounded by the persistent migration of skilled health professionals to high-income nations [4–9]. The unprecedented levels of international migration of health workers in the past few decades prompted the unanimous adoption, on May 21 2010, of the WHO Global Code of Practice on the International Recruitment of Health Personnel by all 193 WHO member states convening the Sixty-third World Health Assembly [10]. Designed as a multilateral framework to mitigate health personnel migration and its negative effects on health systems [11–12], the WHO Code of Practice (CoP) purports to define ethical standards for the recruitment of migrant health workers “in a manner that strengthens the health systems of developing countries, countries with economies in transition and small island states” [10]. It urges high-income destination countries to consider the special circumstances and unmet needs of low- and middle-income countries (LMICs) experiencing net emigration and severe health workforce shortages, and it challenges all countries to address their health disparities and staffing needs with their own domestic resources.

Additionally, the CoP advocates “circular migration” as a partial solution to health personnel emigration, enabling health worker émigrés to return periodically to their native countries to provide healthcare services without losing re-entry privilege in their adopted countries. It is noteworthy that circular migration may be hindered in both directions. Through visa restrictions, a “doctor-receiving country” like the US may make it difficult for US-based migrant health workers from LMICs (e.g., India, Pakistan, the Philippines, Nigeria, or South Africa) to shuttle between the US and their home countries, unlike citizens of most Western nations who can travel on short-term visits (up to 90 days) to the US without visas [13].

The “sending countries”, the potential beneficiaries of circular migration, may also hinder émigré physicians’ return to their native countries by preventing them from retaining their native nationality when they accept citizenship in the country of immigration [14]. In 2009, only 25 of 54 African countries allowed dual citizenship for their nationals living abroad [14]. In a world of increased transnationalism [15–16], such restrictions limit the reengagement of émigrés in their native countries, making it difficult for them to travel home without visa requirements, and to participate in trade, investment, knowledge exchange, and health technology transfer with their home countries [14].

Although the initial implementation of the CoP has been disappointing [17–18], its aims reflect a universal awareness of the destabilizing effects of skilled migration on the development and health systems of many LMICs [8–9]. At the time, officials of the US Health Resources and Services Administration (HRSA) and the US Office of Global Affairs observed: “Due to the privatized nature of the US health care and health personnel recruitment systems, our country will face challenges in the implementation of the WHO Code of Practice” [19]. Such a statement raises questions as to whether a non-binding policy to address health workforce migration can be effective in a global, often privatized context, where many pledges of aid and other good intentions on the part of wealthy countries may go unfulfilled [20].

Moreover, the CoP does not explicitly define “international recruitment” [21]. Thus, conflicting interpretations may discourage the “active” recruitment of health personnel from LMICs while condoning its “passive,” more pervasive form. This passive recruitment of health personnel from LMICs has been a modus operandi of the US, which recruits international medical graduates (IMGs) through the channel of its graduate medical education (GME) residency training programs [22–24]. Each year in the US, following the traditional National Residency Match Program (NRMP) that assigns most US senior medical students to their preferred residency/specialty choices, there are thousands of unfilled residency positions that are then filled by IMGs [25]. Of note, a growing number of IMGs entering US residency training are in fact US citizens who attended medical schools overseas (mainly in the Caribbean islands) [26]. However, as shown in Table 1, foreign nationals are still the numerical majority of IMGs participating and obtaining residency positions through the NRMP [27–30].

**Table 1:** Numbers and percentages of US and non-US citizens who graduated from international medical schools in the National Residency Match Program after the 2010 launch of the CoP.

	US IMGs		Non-US IMGs		Total matched		
	Participants (n)	Matched (%)	Participants (n)	Matched (%)	IMGs (n)	US IMGs (%)	Non-US IMGs (%)
2014 Match	5,133	2,722 (53%)	7,334	3,633 (49.5%)	6,355	42.8%	57.2%
2013 Match	5,095	2,706 (53.1%)	7,568	3,601 (47.6%)	6,307	42.9%	57.1%
2012 Match	4,279	2,102 (49.1%)	6,828	2,775 (40.6%)	4877	43.1%	56.9%
2011 Match	3,769	1,884 (50.0%)	6,659	2,721 (40.9%)	4,605	40.7%	58.9%
Total	18,276	9,414 (51.5%)	28,389	12,730 (44.8%)	22,144	42.5%	57.5%

Note: CoP, WHO Global Code of Practice on the International Recruitment of Health Personnel; US IMGs, citizens of the US who graduated from non-US medical schools; non-US IMGs, foreign nationals who graduated from non-US medical schools.

Data sources: Educational Commission for Foreign Medical Graduates [27–30].

Both IMGs and US medical graduates (USMGs) must obtain a medical license to practice medicine in the US. While each US state issues medical licenses under its own rules and requirements, a common feature is that every applicant physician must complete a US GME residency training program, typically of three years duration, longer for surgical disciplines. This residency requirement applies even for IMGs who have already completed such post-graduate specialization training in their home countries. Before applying for admission into US GME residency training, IMGs must be certified by the Educational Commission on Foreign Medical Graduates (ECFMG), the independent organization tasked with assessing and certifying IMGs’ readiness to begin residency and pursue licensed practice in the US [31]. Many IMGs do not succeed to obtain the ECFMG certification for admission into US residencies [31], and many ECFMG-certified IMGs do not obtain a residency slot [27–30]. Despite these hurdles and the possibility of failure, many IMGs are continuously lured into the US by the perceived benefits provided by US medicine such as high income, excellent conditions of service, state-of-the-art equipment and research facilities, steady stream of funding for research, and opportunities for family members [32–39]. Hence, many foreign physicians migrate, but fail to obtain a license to practice medicine in the US.

This “brain waste” [40–42] of IMGs in the US workforce (doctors who cannot practice medicine) is hard to quantify because no comprehensive database system devoted to these unlicensed physicians exist in the US, unlike their licensed counterparts whose biographic records are collected every year through the American Medical Association Masterfile Physician Professional Data (AMA Masterfile) [43]. The ECFMG is the organization most likely to possess a fair amount of data on unlicensed IMGs residing in the US. However, the ECFMG application and certification data are proprietary and inaccessible to external users. Thus, in this study, we describe “international recruitment” as the admission of IMGs and foreign-born nationals into the US physician workforce. Owing to restricted access to ECFMG data, our definition includes only SSA migrant physicians who are in licensed practice or in residency training in the US.

Data monitoring and information sharing on health personnel migration are vital to build the evidence base necessary for evaluating the effectiveness and relevance of the CoP whose first comprehensive review is scheduled in May 2015 during the 68^th^ World Health Assembly [10]. In keeping with these two key recommendations, we sought to monitor the post-CoP migration of physicians originating from Sub-Saharan Africa (SSA), the region of greatest need, and recruited into the physician workforce of the US. We chose the US as the country with the largest global stock of IMGs in its workforce [44–45]. We captured all SSA immigrant physicians in residency or licensed practice in the US three years post-adoption of the CoP. We then described their growth rates, location patterns, and projected numbers in 2015.

## Methods

### Defining SSA Migrant Physicians

We defined “SSA migrant physicians” or “SSA-origin physicians” as: a) US-based IMGs who graduated from schools located in the SSA region; and b) US-based SSA natives (i.e., SSA-born) who graduated from medical schools located in the US or in other non-SSA foreign nations such as India, Dominica, Grenada, Sint Maarten, or the UK [7], [37]. We designated migrant physicians in the former group “SSA-trained” (SSA-IMGs), and labeled those in the latter group “SSA-born, but foreign-trained”.

While some SSA-born, but foreign-trained physicians may have left their native countries as children or teenagers, and have completed their medical education abroad with no support from their native governments, others may have been funded in part or in whole by their native governments with the expectation that they will return home to practice after completing their training abroad [46]. A 2004 study identified 11 SSA countries with no medical school and 24 SSA countries with only one medical school at the time [4]. The University of Botswana School of Medicine, the only medical school in Botswana, graduated the first Botswana-trained medical doctors in 2014 [47–49]. The University Namibia School of Medicine, the only medical school in Namibia, admitted its first students in 2010 [50]. Hence, through necessity, several SSA countries have sent many of their nationals abroad over the years for medical education and specialization.

Because SSA migrant physicians admitted into US residencies after May 2010 would be expected to have spent at most three years in residency training by December 2013, we defined “post-CoP recruits” as US-based SSA-origin physicians in first through third residency years as of December 2013. We compared their number to “pre-CoP recruits”—US-based SSA licensed physicians and SSA resident physicians beyond their third year in residency training as of December 2013.

### Data

Aggregate data on SSA-origin physicians were collected in December 2013 from the medical database system of an AMA Masterfile licensee [51]. The AMA Masterfile is the most comprehensive biographic database of all US-based licensed and resident physicians, including US medical graduates (USMGs) and IMGs, and physicians who are and are not AMA members [52]. Some concerns vis-à-vis the AMA Masterfile are worth mentioning. The birth country variable in the AMA Masterfile contains a very large proportion of missing data values [4], [7], [45]; 70% of SSA-IMGs in the 2011 AMA Masterfile did not report their country of birth [7]. Moreover, the AMA Masterfile is proprietary, and its data are expensive to access. This limits data access for researchers interested in analyzing and publishing physician workforce data, a *sine qua non* for CoP compliance.

While an unknown number of IMGs appearing in the AMA Masterfile may return to their countries of origin after residency training, the very large number of foreign-born and foreign-educated physicians found in the US physician workforce reflects a preference to stay and practice in the US after residency [53]. Thus, we considered all SSA-born and SSA-educated resident physicians admitted into the US physician workforce as émigrés. We included in our analysis only those physicians reporting “active” or “semi-retired” (working <20 hours per week) status in the December 2013 AMA Masterfile.

Within the context of a multifaceted study of SSA-to-US physician migration [37], we used available data from the 2002 [4] and 2011 [7] AMA Masterfiles as baseline metrics for the estimation of physician emigration rates. To estimate the number of SSA migrant physicians in the AMA Masterfile in 2015, we extracted aggregate residency completions for active SSA-origin physicians from 2000 to 2012 in the 2013 AMA Masterfile. We then projected three additional years to 2015 with linear regression, assuming a balance of additions (i.e., incoming residents) and drop-outs (i.e., inactive and retired physicians).

### Geo-spatial Analysis

IMGs are distributed across the US with certain geographic patterns. For example, many IMGs are recruited into the US physician workforce with the expectation that they may serve in rural and medically underserved areas [54]. Although the evidence suggests that this expectation is only partially met [55–57], this was the rationale for the initial adoption and subsequent extensions of the US Immigration Service Conrad 30 Waiver program which gives H1B nonimmigrant status to non-US IMGs on exchange visa (J-1) if they work at a health care facility located in an area designated as a “Health Professional Shortage Area,” “Medically Underserved Area,” or “Medically Underserved Population” [58–60]. Accordingly, we sought not only to quantify the number and growth rate of SSA migrant physicians in the US, but also to determine their location patterns. We aggregated SSA-origin physicians’ residential and professional addresses by zip codes and converted zip codes into global positioning system coordinates using GPS Visualizer [61]. We then applied ArcGis [62], a geographic information system, to analyze and visualize geocoded data.

## Results

### Sub-Saharan African Migrants in the 2013 US Physician Workforce

In the December 2013 AMA Masterfile (N = 940,456), there were 11,787 active and semi-retired SSA-origin physicians. This total represents ≈1.3% of the 2013 US physician workforce, and includes both physicians who graduated from SSA-based medical schools (SSA-IMGs), and physicians who were born in SSA, but graduated from non-SSA medical schools. Of the above total, 19.4% (n = 2,295) graduated from US medical schools, 68% (n = 8,003) were SSA-IMGs, and 12.6% (n = 1,489) were SSA-born IMGs graduated from international medical schools located outside the SSA region. SSA-origin IMGs in the 2013 AMA Masterfile (n = 9,492) represented 3.7% of the total number of IMGs (N = 256,739) (Table 2), and 4.4% of all non-US IMGs (N = (256,739–42,007) = 214,732) respectively.

**Table 2:** Medical graduates^a^ from Sub-Saharan Africa (SSA), the United States, and elsewhere in the December 2013 American Medical Association (AMA) Physician Masterfile.

**International medical graduates (IMGs)**	n	Percent of subtotal	Percent of total
SSA-origin IMGs	9,492	3.7%	1.1%
Graduates of SSA medical schools (SSA-IMGs)^**b**^	8,003	3.1%	0.9%
SSA-born graduated from non-SSA international medical schools	1,489	0.6%	0.2%
US IMGs^**c**^	42,007	16.4%	4.5%
Other IMGs^**d**^	205,240	79.9%	21.8%
Subtotal	256,739	100%	27.3%
**US medical graduates (USMGs)**			
SSA-born graduated from US medical schools	2,295	0.3%	0.2%
US-born graduated from US medical schools	577,336	84.5%	61.4%
Other USMGs^**e**^	104,086	15.2%	11.1%
Subtotal	683,717	100%	72.7%
**Total**	940,456		100%

Note:

^**a**^ Include only active and semi-retired physicians (i.e., physicians working less than 20 hours a week)—about 6.5% (62,507) of active and semi-retired physicians in the 2013 AMA Masterfile were >70 years old.

^**b**^ We did not detail the proportions of SSA-born vs. non-SSA-born SSA-IMGs because over two thirds of SSA-IMGs found in the AMA Masterfile do not report their birth countries [4], [7]. However, in our previous analysis of the 2011 AMA Masterfile [7], we reported that ≈16% of SSA-IMGs with complete birth country data were born in the US and in other non-SSA nations. Although we did not perform a systematic analysis of their surnames, we suspect that the majority of SSA-IMGs among this small minority of foreign-born are offspring of African immigrants who were living abroad at the time of their children’s birth, and returned to their countries of origin to raise them.

^**c**^ US IMGs, Citizens of the US who graduated from non-US medical schools.

^**d**^ Other IMGs include non-SSA IMGs, non-US IMGs, and all IMGs with missing birth country data. Some SSA-born physicians educated outside SSA and outside the US but with missing birth country data may be in this group. It is also possible that there are some US IMGs with missing birth country data in this group.

^**e**^ Other USMGs include all non-SSA foreign-born physicians graduated from US medical schools, and all potential USMGs with missing birth country data. Some SSA-born USMGs with missing birth country data may be in this group.

Data source: Redi-Med Data Interactive Medical Database System [51].

**Fig 1:**
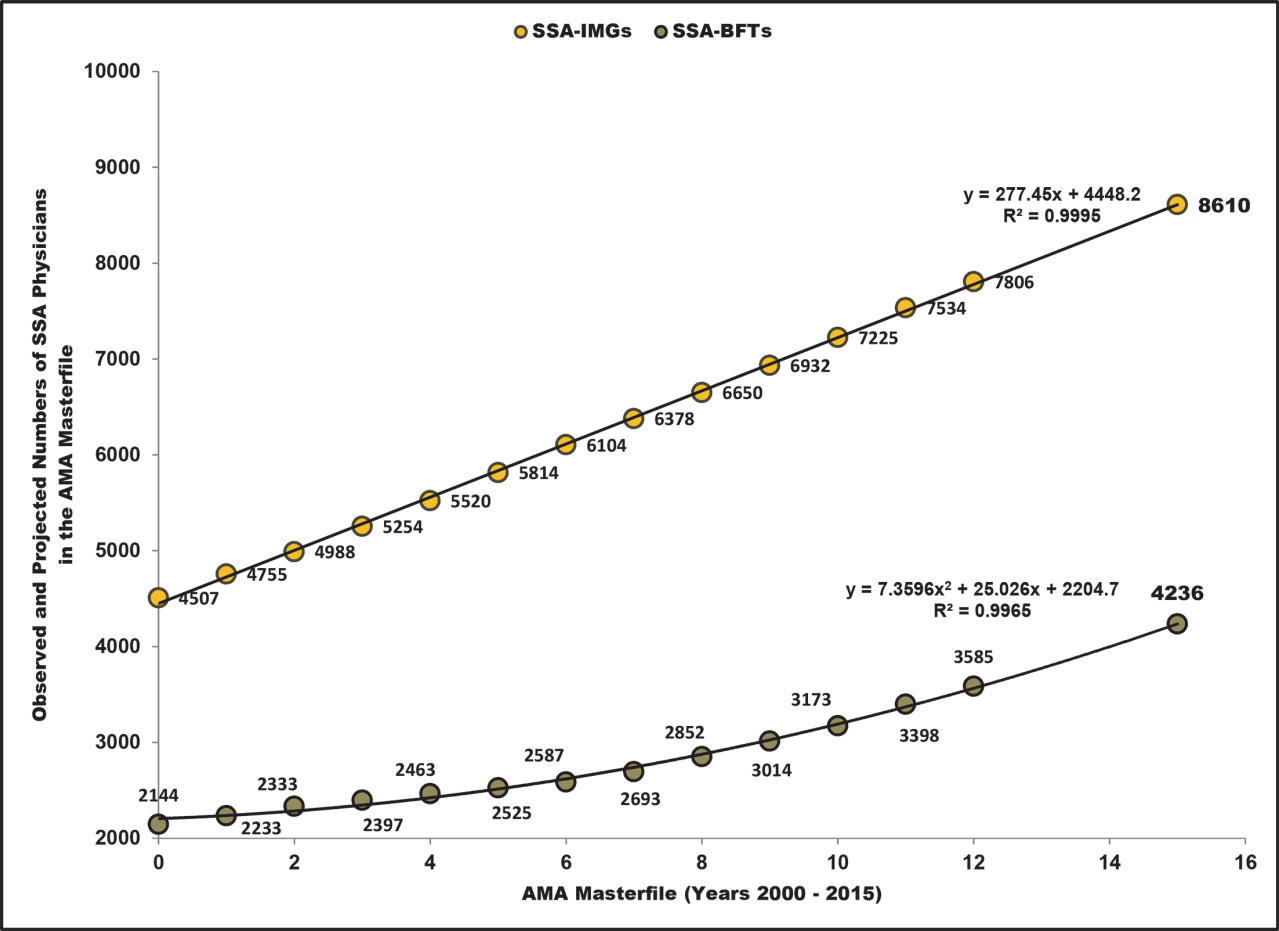
Projected numbers of active and semi-retired Sub-Saharan African (SSA) migrant physicians in the 2015 American Medical Association (AMA) Physician Masterfile. Note: Based on available residency completions and expected completions of active and semi-retired Sub-Saharan African migrant physicians in the 2013 AMA Physician Masterfile [27]; SSA-IMGs, international medical graduates trained in SSA-based medical schools; SSA-BFTs, SSA-born, but foreign-trained physicians (including US medical graduates and international medical graduates trained in non-SSA-based medical schools).

Going by their residency status, 15.7% (1,849 out of 11,787) of these SSA migrant physicians entered the US physician workforce after the May 2010 adoption of the CoP, reflecting a post-CoP annual growth rate of 5.3%. Compared to pre-CoP annual growth rate (3.6% from 2002 to 2010), post-CoP residency admission trends are up for SSA-origin physicians (Table 3). As suggested by Fig 1, this overall increase is driven mainly by the growing number of SSA-born, but foreign trained physicians (i.e., attending medical schools outside SSA).

**Table 3:** Sub-Saharan African (SSA) immigrant physicians appearing in the American Medical Association (AMA) Physician Masterfile before and after the launch of the WHO Global Code on the International Recruitment of Health Personnel (CoP).

	2002 AMA data	2013 AMA data (active & semi-retired physicians)			Pre-CoP recruitment growth rate (2002–2010)		Post-CoP recruitment growth rate (2010–2013)	
	Baseline data^a^	Pre-CoP recruits	Post-CoP recruits	Subtotal	Overall percent increase	Annual recruitment growth rate	Overall percent increase	Annual recruitment growth rate
Graduates from SSA medical schools (SSA-IMGs)	5,334	6,896	1,107	8,003	29.3%	3.9%	16.1%	4.6%
SSA-born graduates from medical schools outside SSA and the United States	1,041	1,230	259	1,489	18.2%	2.4%	21.1%	6.2%
SSA-born graduates from US medical schools	1,455	1,812	483	2,295	24.5%	3.3%	26.7%	7.6%
Total	7,830	9,938	1,849	11,787	26.9%	3.6%	18.6%	5.3%

Note: Post-CoP recruits, physicians in first through third residency years as of December 2013; Pre-CoP recruits, licensed and resident physicians beyond their third year of residency training as of December 2013; Semi-retired physicians, physicians working less than 20 hours a week; SSA-IMG, international medical graduate who completed medical school in the SSA region; Annual pre-CoP recruitment growth = 2002–2010 percent increase divided by 7.5; Annual post-CoP recruitment growth rate = 2010–2013 percent increase divided by 3.5.

^**a**^ Baseline data sources: Hagopian et al. [4]; Tankwanchi [37]; Redi-Medi Data Interactive Medical Database System [51].

**Fig 2:**
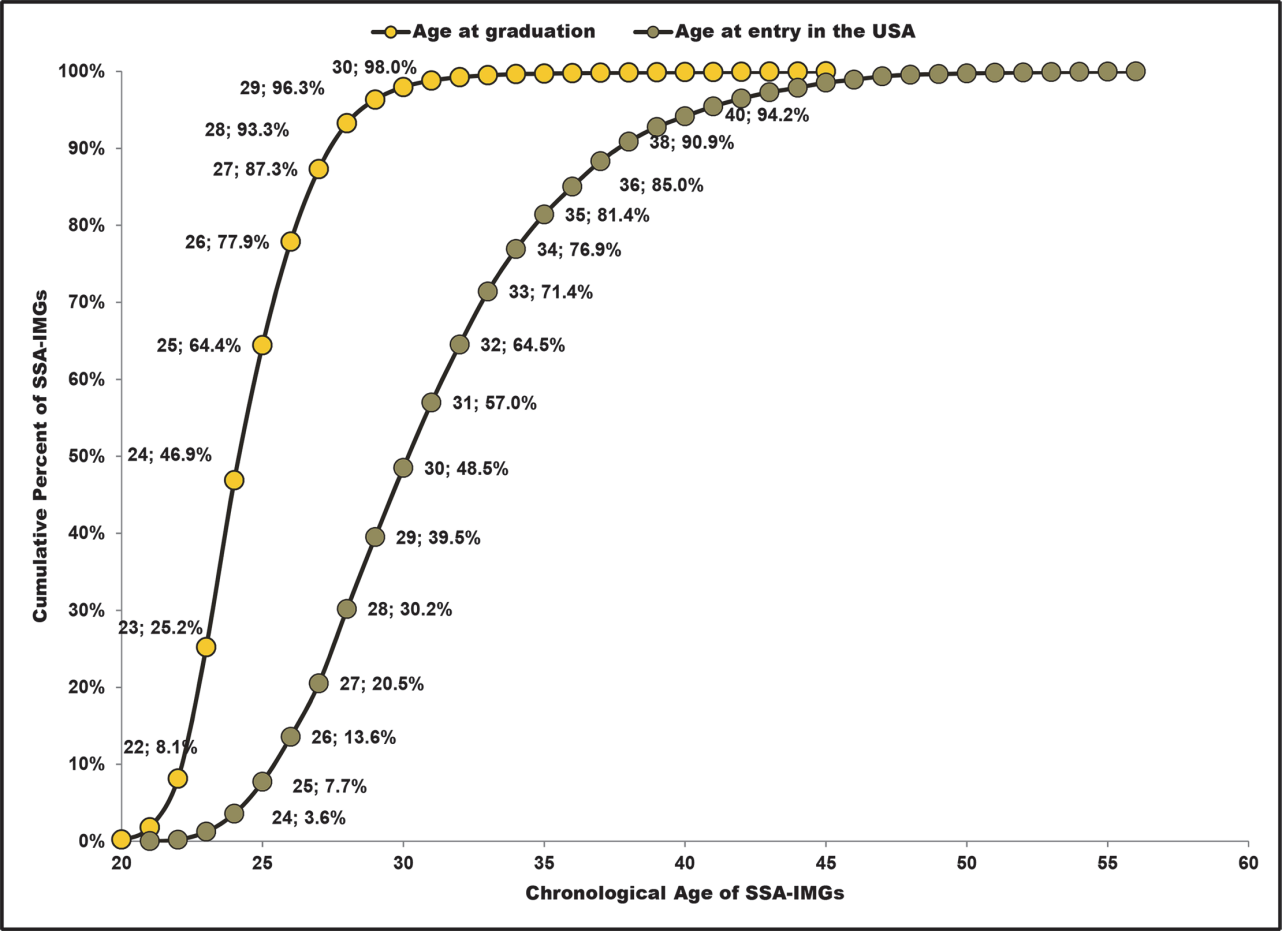
Cumulative distribution curves for Sub-Saharan African international medical graduates’ (SSA-IMGs) ages at time of graduation and at time of entry into the United States. Data source: From the 2011 American Medical Association Physician Masterfile data in Tankwanchi [37].

Extrapolating yearly residency completion data to 2015 yielded respectively 8,610 SSA-IMGs and 4,236 SSA-born, but foreign-trained physicians (Fig 1). This suggests that, when WHO member states convene in May 2015 at the 68th World Health Assembly to report their progress vis-à-vis the CoP implementation, there will be on aggregate 12,846 active physicians originating from the SSA region in the US physician workforce. Of these émigrés, 22.7% (2,908 out of 12,837) will represent post-CoP residency admissions. The trend towards younger migrants is apparent; >80% of migrant physicians graduating from SSA schools are estimated to enter the US by age 35 (Fig 2).

### Migrants’ Locations

**Fig 3:**
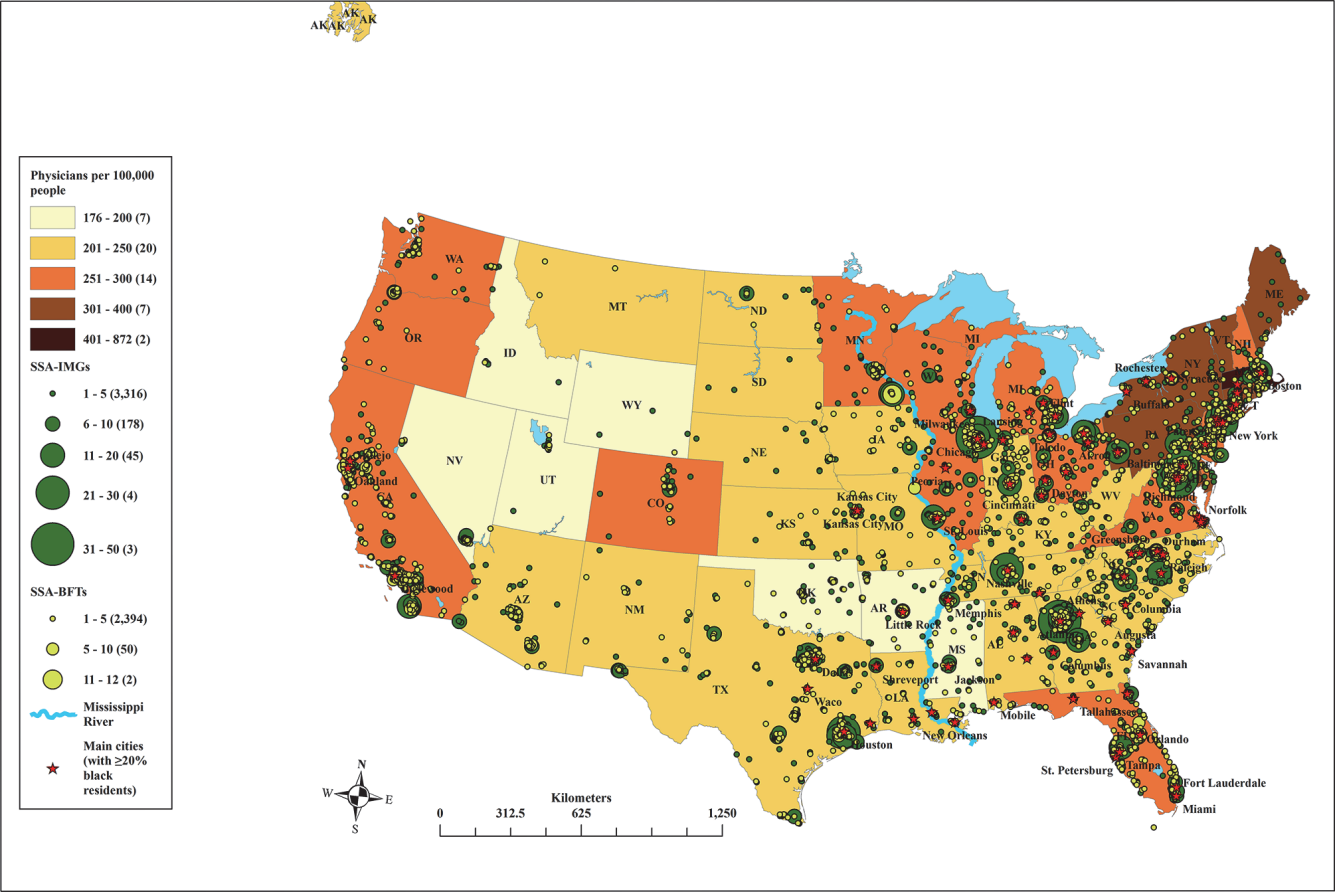
Geography of loss: Spatial distribution of Sub-Saharan African migrant physicians across the United States. Note: SSA-IMGs, international medical graduates trained in Sub-Saharan African-based medical schools; SSA-BFTs, Sub-Saharan African-born, but foreign-trained physicians (i.e., graduates of US and other non-SSA foreign medical schools). Data sources: Redi-Med Data Interactive Medical Database System [51]; Environmental Systems Research Institute [62]; Association of American Medical Colleges [63].

Five main observations are noted vis-à-vis SSA migrant physicians’ locations in the US. 1) They locate predominantly in metropolitan areas east of the Mississippi River; except for populous Texas and California, western venues receive comparatively fewer SSA migrant physicians (Fig 3). 2) Central and Mountain states with some of the lowest physician densities (e.g., Idaho, Nevada, Utah, and Wyoming) attract the lowest numbers of SSA migrant physicians (Fig 3). 3) Higher racial diversity of the city seems to serve as a magnet for SSA migrant physicians; more locate to urban areas with the proportion of the black population exceeding 20% (Fig 3).

**Fig 4:**
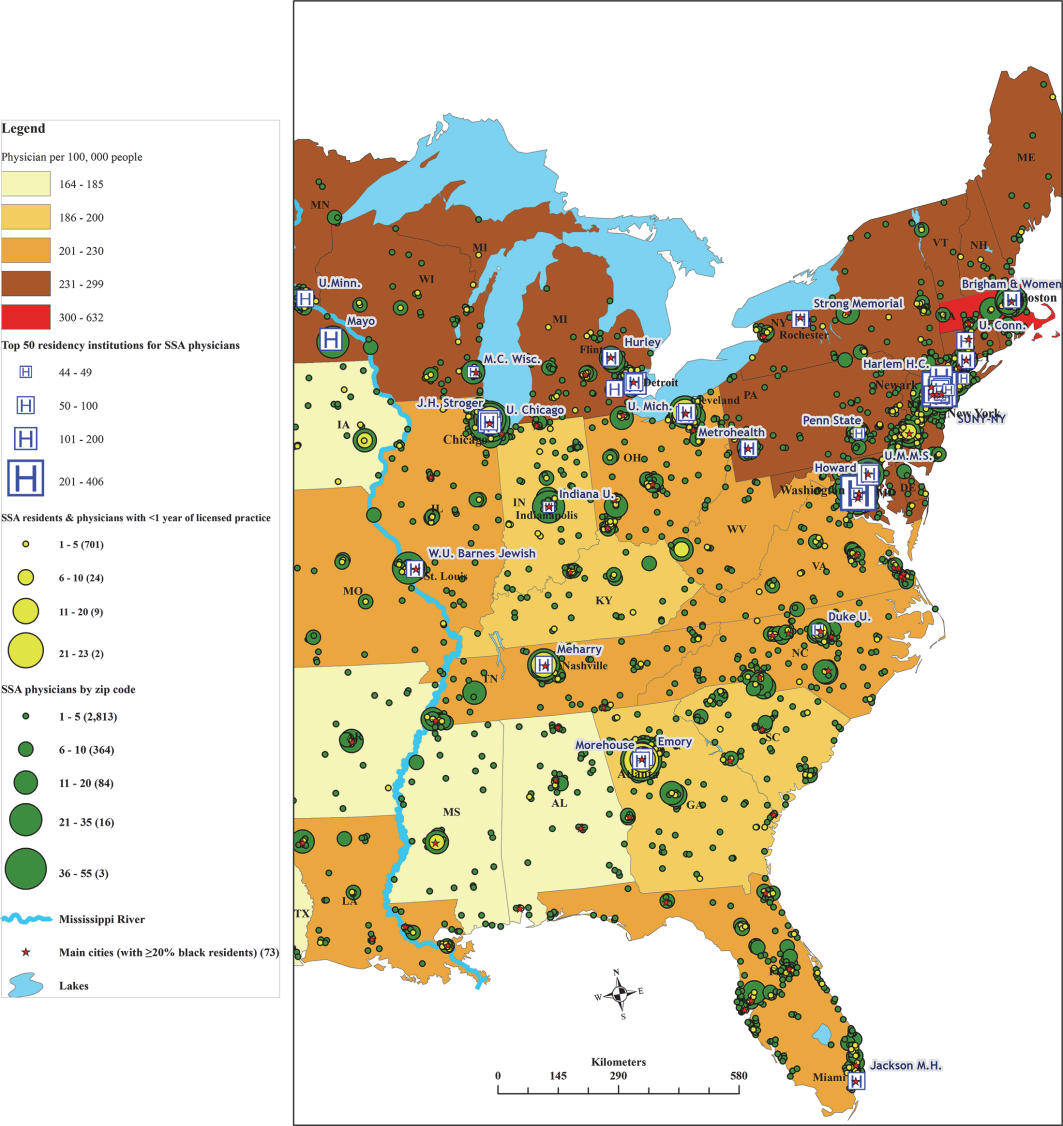
Main residency institutions of Sub-Saharan African migrant physicians in the Eastern Region of the United States. Note: Full names of residency programs appearing on the map are provided as supporting information (S1 Table). Data sources: Redi-Med Data Interactive Medical Database System [51]; Environmental Systems Research Institute [62]; Association of American Medical Colleges [63].

4) As shown in Fig 4, recent SSA migrant physicians, including post-CoP recruits, are found primarily in the same zip codes as their earlier immigrant counterparts. This clustering of most recent and pioneer SSA migrant physicians in the same geographic location has created sizable migration fields of SSA physicians in New York, Chicago, Atlanta, and the Baltimore-Washington, DC region (Fig 3). 5) These cosmopolitan cities also have some of the most accessible residency institutions for SSA migrant physicians, most notably Howard University Hospital (Washington, DC), and Harlem Hospital Center (New York), both of which draw large numbers of SSA migrant physicians into their residency programs (Fig 4).

## Discussion

Three full years post-adoption of the CoP, evidence from the 2013 AMA Masterfile reveals an increasing number of SSA-origin physicians have been recruited into the US physician workforce, with projections for an even further acceleration as new entrants move through the pipeline while less time is spent in practice in SSA after medical school graduation by the more recent migrant doctors [7], [37]. We found no evidence that the CoP has slowed SSA-to-US physician migration. Instead, we observed a clustering of pre- and post-CoP recruits in the same localities, creating sizeable SSA physician migration fields in several US metropolitan areas, most notably Atlanta, Chicago, Baltimore-Washington, DC, and New York City. The settlement patterns of SSA physicians across the US are consistent with various migration theories, mainly at the meso-level. These include the early concept of “chain migration” and its recent substitute/corollary, “migrant networks.” These two concepts convey the idea that migration is a path-dependent and self-sustaining process whereby earlier migrants facilitate the inflow and adaptation of recent/prospective migrants through social support networks entailing informational, cultural, financial, and material assistance [64–65].

Some of the African doctors appearing in the AMA Masterfile after May 2010 may have entered the US before the adoption of the CoP, thereby inflating post-CoP estimates. At the same time, among physicians who immigrated to the US after the CoP was adopted, some may still be completing pre-residency admission requirements, and therefore are not yet captured by the AMA Masterfile, thus deflating post-CoP estimates. Since the AMA Masterfile contains a high rate of missing data on birth country [4], [7], [45], we may be undercounting physicians who were born in SSA but trained in the US and in other non-SSA countries. Moreover, there is often a time lag for data entry and status change in the AMA Masterfile, again undercounting SSA physicians. Finally, as observed in the introduction, unlicensed doctors do not appear in the AMA Masterfile, an underestimate of migration.

Hence, our emigration figures are very conservative estimates. Yearly replications of our analysis would be helpful in confirming our impressions of the limited policy impact of the CoP vis-à-vis SSA-to-US migration. As a matter of CoP compliance, it may be necessary for HRSA and the US Office of Global Affairs, authorities charged with the implementation of the CoP in the US, to issue requests for proposals to academics and relevant organizations to conduct robust studies on health workforce migration in the US. Similar studies could be conducted in other high-income nations to provide a more complete picture of the SSA physician “brain drain,” as well as within the African continent (e.g., Mozambican or Zambian doctors migrating to South Africa).

The spirit of the CoP, by all appearances, has not permeated the ethos of some of the most influential health organizations that encourage IMGs’ migration to the US. The AMA [66], the American College of Physicians [67], the American College of Surgeons [68], the American Hospital Association [69], the Association of American Medical Colleges [70], and the National Rural Health Association [71], combining a membership of over 780,000 individuals and more than 6,000 hospitals and health systems, have all endorsed the 2012 Conrad State 30 Improvement Act and the 2013 Conrad State 30 and Physician Access Act [59]. These bills intended to make permanent the Conrad State 30 J-1 Visa Waiver Program that has been law for 20 years. While the bills failed to pass, the successful reauthorization in 2013 of the Conrad State 30 J-1 Visa Waiver Program [60] indicates a highly permissive status quo in US policy vis-à-vis its recruitment of IMGs from LMICs. We speculate that this is perceived to be less costly than expansion of the US National Health Service Corps to meet health workforce needs with indigenous medical and osteopathic school graduates [72–73].

Although unanimously ratified by all WHO member states, the non-binding nature of the CoP reflects an all-too-rhetorical and reactionary policy [17], preserving the interests of doctor-receiving countries while denying any serious leverage to source countries that lose their health personnel. In high-income countries like the US or Canada, immigration policy is integral to national economic policies and favors highly skilled immigrants [74–75]. It is naïve to expect that such countries may fully implement a discretionary code that could compromise their economic and health care interests.

Without the CoP, one might argue, even more SSA physicians might have migrated. However, data limitations stymie our ability to fully evaluate the effectiveness of the CoP. Indeed, it is very challenging for researchers to monitor the migration and recruitment of foreign health personnel into the US health workforce without unrestricted access to the US Department of State visa application records for foreign health workers, and to proprietary data sources such as the ECFMG application and certification databases and the AMA Masterfile. Moreover, the CoP does not explicitly discourage health workers migration. As we have observed previously [21], the CoP instead empowers migrant health personnel, reasserting their fundamental right to emigrate and to be treated equally to domestically-trained health personnel in the host country.

Due to a payment cap on Medicare-supported residencies that the US Congress imposed in the Balanced Budget Act of 1997, US medical education enrollments have grown faster in recent years than GME positions [76–77]. The opening of 16 new US MD-granting schools matriculating their inaugural classes since 2002, and the expansion of first-year enrollments in existing US medical schools have contributed significantly to this growth [78–79]. If these trends persist, the number of residency slots available to IMGs will decrease significantly and competition to match to specialties will be even fiercer. This potential outcome, in development long before the advent of the CoP, may reduce SSA-IMGs’ admissions into US residencies, and over time, may effectively curb their migration to the US. However, with a physician demand projected to grow by 86,700 to 133,200 in the US by 2025 after the enactment of the Affordable Care Act [80], it is reasonable to anticipate a higher demand for IMGs, especially if the repeated calls by the Association of American Medical Colleges and its allies to increase residency training and funding are heeded [77], [81–82].

Although comprising only 1.3% of the US physician workforce, SSA migrant physicians found in the December 2013 AMA Masterfile represent a significant loss for the health systems in the SSA region. Compared to SSA countries, populous source countries with a tradition of medical migration like India, Pakistan, and the Philippines have much larger numbers of émigré physicians in doctor-receiving countries like the US, the UK, Canada, or Australia [44]. But, relative to the number of physicians remaining in the source countries, the SSA region as a whole has a much higher migration proportion, losing between 13.9% [44] and 28% [6] of its physicians.

The 11,787 SSA émigrés in the December 2013 AMA Masterfile exceed the aggregate number of physicians (N = 11,519) reported in the WHO database [83] in a total of 34 SSA countries—from Liberia (n = 51) to Zambia (n = 836)—with a combined 2012 population approaching 270 million. As we have observed previously [7], [37], if only half of the Liberian physicians licensed in the US were to return to Liberia to practice, they could more than double the Liberian physician workforce. With the deadliest Ebola epidemic (2014–2015) wreaking havoc on the very weak health systems of Liberia, Sierra Leone, and Guinea [84–86], the service of such doctors would be especially valuable.

The WHO database [83] indicates that there were a total of 103 physicians in Liberia in 2004, but only 51 physicians in 2008, a 50.5% total physician loss within four years. We do not have the most current counts of physicians available in Liberia because they have not been updated in the WHO database since 2008. But, we do know that the current Ebola epidemic has further depleted Liberia’s meager health workforce. The *Ebola Situation Report* of March 18, 2015 indicates that 180 out 372 health workers infected in Liberia have died from the Ebola virus disease [86].

As the epidemic ravaged her resource-constrained country in October 2014, Liberia’s president, Ellen Johnson Sirleaf, made an impassioned appeal to the global community (including Liberia’s medical diaspora) to mobilize more human, financial, and material resources to help Liberia, Sierra Leone, and Guinea fight the epidemic [87]. We do not know how many Liberian émigré physicians have answered the call, and have gone back to Liberia to assist. But, the decision of President Sirleaf’s own physician son, Dr. James Adama Sirleaf, to stay in the US leads one to suspect that most Liberian physician émigrés stayed away from their home country during the Ebola crisis [88]. The Georgia-based emergency physician, observed: “The symbolism of me going there and potentially getting Ebola when I have a nine- and a seven-year-old at home isn't worth it just to appease people. I’ve made a commitment not to live in Liberia for many reasons, and I think my contribution means more” [89]. Although controversial, Dr. Sirleaf’s decision speaks to the limitation of promoting circular/return migration as a long-term policy option to address the SSA health workforce shortage, especially in times of crisis.

Understandably, many SSA physicians aspiring for medical specializations that are unavailable or limited in their home countries will likely seek such specializations abroad while they are still relatively young. This may explain in part why over 80% of SSA-IMGs appearing in the AMA Masterfile entered the US by age 35 (Fig 2). Thus, interventions aimed at strengthening health systems and promoting health workforce retention in SSA [8], [85], [90–92] should emphasize early-career physicians 35 years old or younger. Discouraging their early migration and incentivizing their practice while expanding medical specialization opportunities in-country and regionally will be necessary steps toward greater retention of home-grown physicians. Stronger measures than the current CoP aimed at receiving countries are also necessary, however. Without such measures, SSA countries will never be able to compete, particularly with the US and other high-resource countries.

## Conclusion

The SSA-to-US physician “brain-drain” represents a paradox in US national policy. In many African countries, US health investments have dwarfed those of other donor nations in recent years, helping decrease the burden of infectious diseases and extend life expectancy [93–94]. An important component of the US President’s Emergency Plan for AIDS Relief (PEPFAR) is the support of health workforce training in Africa through such initiatives as the Nursing Education Partnership Initiative (NEPI), and the Medical Education Partnership Initiative (MEPI), the Consortium of New Southern African Medical Schools (CONSAMS), and the Southern Africa Consortium for Research Excellence (SACORE) [94–99]. The August 2014 supplement of *Academic Medicine* comprises 33 articles entirely devoted to the analysis of MEPI [100]. This reflects recognition of urgency to scale up education and training of health workers in SSA, and the priority given to it by the US government. Yet, supporting such African health workforce capacitation efforts works at cross-purposes when failing to address the passive, yet pervasive recruitment of African physicians into the US workforce.

## Supporting Information

S1 Table Full names of residency programs appearing in Fig 4. (DOCX)
