# Supplementary material for: Monitoring Sub-Saharan African Physician Migration and Recruitment Post-Adoption of the WHO Code of Practice: Temporal and Geographic Patterns in the United States
Source: PLoS One. 2015 Apr 13;10(4):e0124734. doi: 10.1371/journal.pone.0124734 (PMC4395332; doi:10.1371/journal.pone.0124734)
Supplement: S1 Table — (DOCX) [file pone.0124734.s001.docx]

**S1 Table. Full names of residency institutions appearing in Fig. 4**

| **Short name** | **Full name** |
| --- | --- |
| Brigham & Women | Brigham and Women's Hospital |
| Duke U. | Duke University Medical Center |
| Emory | Emory University School of Medicine |
| Harlem H.C. | Harlem Hospital Center |
| Howard | Howard University Hospital |
| Hurley | Hurley Medical Center |
| Indiana U. | Indiana University School of Medicine |
| J.H. Stroger | John H. Stroger Jr. Hospital of Cook County |
| Jackson M.H. | Jackson Memorial Hospital - Jackson Health System |
| M.C. Wis. | Medical College of Wisconsin Affiliated Hospitals |
| Mayo | Mayo School of Graduate Medical Education - Mayo Foundation |
| Meharry | Meharry Medical College School of Medicine |
| Metrohealth | Metrohealth Medical Center |
| Morehouse | Morehouse School of Medicine |
| Penn State | Penn State University Hospital - Milton S. Hershey Medical Center |
| Strong Memorial | Strong Memorial Hospital, University Rochester Medical Center |
| SUNY-NY | SUNY Health Sciences Center Brooklyn |
| U. Chicago | University of Chicago Hospitals |
| U. Conn. | University of Connecticut School of Medicine |
| U. Mich. | University of Michigan Hospitals and Health Centers |
| U. Minn. | University of Minnesota Medical School |
| U.M.M.S. | University of Maryland Medical System |
| W.U. Barnes Jewish | Washington University in St. Louis School of Medicine - Barnes Jewish Hospital |
